# Sex Differences in the Effects of COPD on Incidence and Outcomes of Patients Hospitalized with ST and Non-ST Elevation Myocardial Infarction: A Population-Based Matched-Pair Analysis in Spain (2016–2018)

**DOI:** 10.3390/jcm10040652

**Published:** 2021-02-08

**Authors:** Javier de-Miguel-Diez, Rodrigo Jiménez-García, Valentín Hernandez-Barrera, Zichen Ji, José María de Miguel-Yanes, Marta López-Herranz, Ana López-de-Andrés

**Affiliations:** 1 Pneumology Department, Hospital General Universitario Gregorio Marañón, 28007 Madrid, Spain; jmiguel.hgugm@salud.madrid.org (J.d.-M.-D.); jizich72@gmail.com (Z.J.); 2 Department of Medicine, Faculty of Medicine, Universidad Complutense de Madrid, 28040 Madrid, Spain; 3 Department of Public Health & Maternal and Child Health, Faculty of Medicine, Universidad Complutense de Madrid, 28040 Madrid, Spain; anailo04@ucm.es; 4 Preventive Medicine and Public Health Teaching and Research Unit, Department of Medical Specialties and Public Health, Universidad Rey Juan Carlos, Alcorcón, 28922 Madrid, Spain; valentin.hernandez@urjc.es; 5 Department of Internal Medicine, Hospital General Universitario Gregorio Marañón, 29007 Madrid, Spain; josemaria.demiguel@salud.madrid.org; 6 Faculty of Nursing, Physiotherapy and Podology, Universidad Complutense de Madrid, 28040 Madrid, Spain; martal11@ucm.es

**Keywords:** STEMI, NSTEMI, COPD, sex differences, incidence, in-hospital mortality

## Abstract

We aimed to compare the incidence, clinical characteristics, and outcomes of patients admitted with myocardial infarction (MI), whether ST elevation MI (STEMI) or non-ST elevation MI (NSTEMI), according to the presence of chronic obstructive pulmonary disease (COPD), and to identify variables associated with in-hospital mortality (IHM). We selected all patients with MI (aged ≥40 years) included in the Spanish National Hospital Discharge Database (2016–2018). We matched each patient suffering COPD with a non-COPD patient with identical age, sex, type of MI, and year of hospitalization. We identified 109,759 men and 44,589 women with MI. The MI incidence was higher in COPD patients (incident rate ratio (IRR) 1.32; 95% confidence interval (CI) 1.29–1.35). Men with COPD had higher incidence of STEMI and NSTEMI than women with COPD. After matching, COPD men had a higher IHM than non-COPD men, but no differences were found among women. The probability of dying was higher among COPD men with STEMI in comparison with NSTEMI (odds ratio (OR) 2.33; 95% CI 1.96–2.77), with this risk being higher among COPD women (OR 2.63; 95% CI 1.75–3.95). Suffering COPD increased the IHM after an MI in men (OR 1.14; 95% CI 1.03–1.27), but no differences were found in women. COPD women had a higher IHM than men (OR 1.19; 95% CI 1.01–1.39). We conclude that MI incidence was higher in COPD patients. IHM was higher in COPD men than in those without COPD, but no differences were found among women. Among COPD patients, STEMI was more lethal than NSTEMI. Suffering COPD increased the IHM after MI among men. Women with COPD had a significantly higher probability of dying in the hospital than COPD men.

## 1. Introduction

Chronic obstructive pulmonary disease (COPD) is a major chronic condition with considerable and growing social, health, and economic consequences [1]. Furthermore, its prevalence, morbidity, and mortality are increasing [2]. According to the most recent prevalence study with clinical measurements conducted in Spain, the overall estimated prevalence of COPD among adults aged 40 years or over was 11.8% (95% confidence interval (CI) 11.2–12.5) [3]. Much of the disease burden and healthcare use in COPD patients is related to the management of their comorbidities. The most common associated conditions include cardiovascular disease, skeletal muscle wasting, and stroke [4].

COPD is strongly linked with cardiovascular disease, particularly myocardial infarction (MI). The estimated incidence of MI per year in Spain for the population aged 25 years or over is 2005 per 100,000 for men and 1169 per 100,000 for women [5].

In addition to common risk factors such as smoking and increasing age, some features related to this chronic pulmonary disease, such as systemic inflammation and hypoxia, underlie the pathophysiological interaction between COPD and MI [6]. Consequently, COPD patients have a higher risk of MI than those without COPD. On the other hand, COPD is common in patients with MI, with a prevalence that varies from 7% to 28% [6,7,8]. Moreover, several studies have also found an increased risk of death after MI in COPD patients compared with those without COPD [8,9,10].

Despite ST-elevation myocardial infarction (STEMI) and non-ST elevation myocardial infarction (NSTEMI) having been the focus of intense clinical investigation, scarce data exist on the characteristics and in-hospital mortality (IHM) of patients not enrolled in clinical trials [11]. Furthermore, there is limited information regarding in-hospital adverse clinical outcomes in STEMI and NSTEMI in COPD patients and the existence of a sex difference in these patients [12].

We aimed to compare the incidence, clinical characteristics, and in-hospital outcomes of patients admitted to Spanish hospitals from 2016 to 2018 with a primary diagnosis of MI according to the presence of COPD. We separately analyzed patients with STEMI and NSTEMI and compared the hospital outcomes according to COPD status after matching by MI type, age, and sex. Lastly, we identified the variables associated with IHM for patients with COPD according to sex and MI type.

## 2. Materials and Methods

### 2.1. Study Design and Data Source

We conducted a retrospective observational study. The database for our investigation was the Spanish National Hospital Discharge Database (SNHDD). This database is managed by the Spanish Ministry of Health and includes over 95% of all hospital discharges in Spain, resulting in data of more than 4.2 million discharges each year. Since year 2016, the SNHDD has used the International Classification of Disease version 10 (ICD-10) for coding. This database provides up to 20 diagnoses and 20 procedures for each hospitalization. Details in the SNHDD are available online [13].

### 2.2. Study Population

We analyzed data from all subjects aged 40 years or over collected by the SNHDD in the years 2016, 2017, and 2018.

Our study population included patients discharged with a primary diagnosis of MI (STEMI and NSTEMI) using the specific ICD-10 codes shown in Table S1 (Supplementary Materials). Patients with a code for “subsequent STEMI and NSTEMI (I22.x)” and “other type of myocardial infarction (I21.A.x)” were excluded.

According to the SNHDD, the primary diagnosis is the main clinical condition that resulted in the patient’s hospitalization [13].

The population was stratified according to sex and to the presence or absence of COPD diagnosis codes (J44.0, J44.1, and J44.9) in any diagnosis position (2–20).

### 2.3. Study Variables

The primary outcome variables of interest were the incidence of STEMI and NSTEMI and in-hospital variables such as IHM and length of hospital stay (LOHS). Secondary outcomes of interest were the use of coronary artery bypass graft (CABG) and percutaneous coronary intervention (PCI) during the hospitalization.

Incidences were calculated using the Spanish population with and without COPD estimated with the Spanish National Health Survey 2017 and data provided by the Spanish National Statistics Institute, as described by de Miguel-Diez et al. [14].

Patient-level variables analyzed included age and sex. Comorbidity was assessed with the Charlson comorbidity index (CCI) extracted using the methods for ICD-10 coded administrative databases proposed by Sundararajan et al. [15].

The following conditions and procedures were specifically identified and analyzed: obesity, hypertension, lipid metabolism disorders, renal disease, atrial fibrillation, congestive heart failure, peripheral vascular disease, cerebrovascular disease, dementia, dependence on supplemental oxygen, acute and chronic respiratory failure, sleep apnea, pulmonary hypertension, and mechanical ventilation (see Table S1, Supplementary Materials and [13] for ICD-10 codes).

### 2.4. Matching Method

Men and women with COPD and MI have significantly different ages than non-COPD patients with MI, and an unequal distribution in the type of STEMI, as can be seen in Table S2 (Supplementary Materials). Therefore, in order to control for these confounding factors and to make men and women with COPD more comparable with those without this condition, we matched within the database of the SNHDD each man suffering COPD with a non-COPD man with identical age, type of MI (using all digits in the ICD-10 codes), and year of hospitalization. The same matching method was used for women with and without COPD. If more than one man or woman without COPD was available per man and woman with COPD, the selection was randomly done.

### 2.5. Statistical Analysis

The statistical analysis was conducted separately for women and men.

Descriptive statistics for continuous variables were reported as the mean with standard deviation or the median with interquartile range, whereas counts and percentages were used for categorical variables.

Incidence was analyzed using Poisson regression models, adjusted by age and sex when required, providing incidence rate ratios (IRRs) with 95% confidence intervals (95% CIs) as the measure of association.

Continuous variables were compared using Student’s *t*-test or the Mann–Whitney test. Categorical variables were compared with the chi-square test.

Adjusted odds ratios (ORs) were obtained with multivariable logistic regression to identify variables independently associated with IHM. Models were constructed separately for men and women and according to MI type. Lastly, using the entire database, we analyzed the effect of sex. Details on model construction were previously described [16].

Matching and statistical analysis was conducted with Stata version 14 (Stata, College Station, TX, USA), and significance was set at a two-sided *p*-value of <0.05.

### 2.6. Ethical Aspects

All investigators can apply to the Spanish Ministry of Health for use of the SNHDD, which is provided free of charge [17]. The characteristics of this registry, i.e., anonymized and public access, make it unnecessary to obtain approval from an ethics committee according to the Spanish legislation.

## 3. Results

The numbers of hospital discharges in Spain from 2016 to 2018, for patients aged 40 years or over with a primary diagnosis of MI, were 109,759 (71.11%) men and 44,589 women (28.89%). The overall prevalence of COPD was higher among men than women (7.56% vs. 2.85%; *p* < 0.001).

### 3.1. Incidence of STEMI and NSTEMI According to Concomitant COPD

As can be seen in Table 1, the total incidence of MI was higher *(p* < 0.001) among the COPD population (323.01 per 100,000 persons with COPD) than among those without COPD (198.68 per 100,000 persons without COPD), resulting in an adjusted IRR of 1.32 (95% CI 1.29–1.35). For STEMI, the IRR was 1.09 (95% CI 1.06–1.13), whereas, for NSTEMI, the IRR was 1.61 (95% CI 1.57–1.66).

According to MI type by sex, we found that, among men with COPD, the STEMI incidence was slightly but significantly higher (180.34 vs. 171.00; IRR 1.05, 95% CI 1.02–1.08) and the NSTEMI incidence was clearly higher (201.74 vs. 121.82; IRR 1.65, 95% CI 1.60–1.70) than among men without COPD. Among COPD women, the incidences of STEMI and NSTEMI were also significantly higher than among non-COPD women (IRR 1.46, 95% CI 1.35–1.58 and IRR 1.43, 95% CI 1.32–1.55, respectively).

Men with COPD had a higher incidence of STEMI (180.34 vs. 85.53; *p* < 0.001) and NSTEMI (201.74 vs. 75.29; *p* < 0.001) than women with COPD (Table 1).

### 3.2. Clinical Characteristics and Hospital Outcomes for Men and Women with STEMI According to Presence of COPD

The clinical characteristics and hospital outcomes before and after matching by age and MI type for male patients with STEMI are shown in Table 2.

Men with COPD matched by age and STEMI type had more comorbid conditions than non-COPD men. Remarkably higher was the prevalence of obesity, renal disease, atrial fibrillation, congestive heart failure, peripheral vascular disease, dementia, respiratory failure, sleep apnea, and pulmonary hypertension. As expected, the dependence on supplemental oxygen was higher among COPD men (4.01% vs. 0.15%; *p* < 0.001).

The proportion of COPD men that required mechanical ventilation was significantly higher than matched non-COPD men (9.47% vs. 6.69%; *p* < 0.001). On the other hand, COPD men less frequently received a PCI (54.39% vs. 57.81%; *p* = 0.002).

The median LOHS and the IHM (11.56% for COPD men and 10.01% for non-COPD men; *p* = 0.026) showed significantly higher figures among COPD men after matching.

When we compared women with and without COPD who suffered an STEMI before and after matching, we obtained the results shown in Table 3. As described for men, women with COPD had a significantly higher prevalence of obesity, atrial fibrillation, congestive heart failure, peripheral vascular disease, respiratory failure, sleep apnea, pulmonary hypertension, and dependence on supplemental oxygen.

Regarding procedures and hospital outcomes, we found no significant differences between women with COPD and matched non-COPD women.

### 3.3. Clinical Characteristics and Hospital Outcomes for Men and Women with NSTEMI According to Presence of COPD

As can be seen in Table 4, men with COPD who suffered an NSTEMI had a higher prevalence of all chronic conditions than men matched by age without COPD, except for diabetes, lipid metabolism disorders, and dementia, for which no significant difference was found. COPD men underwent a CABG (2.74% vs. 3.7%; *p* = 0.011) and PCI (37.32% vs. 43.27%; *p* < 0.001) after NSTEMI less frequently than matched non-COPD men.

After matching, men with COPD had a higher LOHS (6.5 vs. 6 days; *p* < 0.001) and IHM (6.82% vs. 5.75%; *p* = 0.039).

The distribution of clinical characteristics and hospital outcomes among women with and without COPD who suffered an NSTEMI is shown in Table 5.

Women with COPD had a significantly higher prevalence of obesity, atrial fibrillation congestive heart failure, respiratory failure, sleep apnea, and dependence on supplemental oxygen.

Regarding the use of procedures during hospitalization, women with COPD received more mechanical ventilation than those without COPD (8.56% vs. 2.68%; *p* < 0.001), with no differences in the use of CABG or PCI. Suffering COPD resulted in a significantly higher LOHS for COPD women.

### 3.4. Multivariable Analysis of IHM

The results of the multivariable logistic regression analysis are shown in Table 6. For men and women with COPD, the risk of dying in the hospital increased with age, congestive heart failure, dementia, acute and chronic respiratory failure, and the need for mechanical ventilation during hospitalization. Renal diseases, atrial fibrillation, and pulmonary hypertension were only risk factors for men suffering COPD.

Undergoing a PCI was associated with a reduction in IHM in all study groups.

Among COPD men, the probability of dying was 2.33 times higher among those with STEMI than among those with NSTEMI (OR 2.33; 95% CI 1.96–2.77). Among women with COPD, this risk was higher (OR 2.63; 95% CI 1.75–3.95).

As can be seen in Table 6, among men, after controlling for study variables, we found that suffering COPD increased IHM after an MI by 14% (OR 1.14; 95% CI 1.03–1.27). However, no significant association was found among women (OR 1.06; 95% CI 0.93–1.38).

Lastly, when we joined the database of men and women with COPD and conducted a multivariable adjustment (Table S3, Supplementary Materials), we found that women with COPD had a significantly higher probability of dying in the hospital than COPD men (OR 1.19, 95% CI 1.01–1.39).

## 4. Discussion

In this Spanish nationwide study comprising 109,759 men and 44,589 women with a primary diagnosis of MI from 2016 to 2018, we found that incidence of STEMI and NSTEMI was higher in men and women with COPD than in those without COPD. In addition, we demonstrated that the incidence of both types of MI was higher among COPD men in comparison with COPD women. Although previous studies have shown that COPD individuals have a higher risk of MI than individuals without COPD [18,19,20], the influence of sex in this relationship was not well elucidated. In addition to sharing common risk factors, COPD itself could be an independent risk factor for MI, possibly mediated through multiple pathways including physiologic and hemodynamic stress due to hypoxia, oxidative stress, endothelial abnormalities, protease/antiprotease imbalance [21], increased systemic inflammation, and reduced FEV_1_ (forced expiratory volume in 1 s) [10].

Men and women with COPD in our study had a higher prevalence of most comorbidities, such as obesity, atrial fibrillation congestive heart failure, respiratory failure, sleep apnea, pulmonary hypertension, and dependence on supplemental oxygen, than matched men and women without COPD. The higher number of comorbidities in patients with MI and associated COPD could have notable repercussions for the management of these patients. Accordingly, physicians could consider COPD patients as high risk for complications, and they could be hesitant when recommending aggressive interventions such as cardiac catheterization and PCI, despite these patients potentially benefiting from the receipt of these evidence-based procedures. Although the factors related to this fact have not been adequately clarified, a potential explanation is that patients with COPD are deemed to be older with higher frailty [6]. It has been described that, at the moment of presentation, COPD patients are less likely to receive PCI compared to those without COPD [7,22]. We also found that men with COPD and STEMI or NSTEMI undergo PCI less frequently than matched non-COPD men. For NSTEMI patients, CAGB was also less frequently used. However, no differences were found among women, possibly due to the small sample size.

Prognosis of patients with COPD after MI was markedly worse compared to patients without COPD. This finding could be explained by the fact that COPD patients might have, in comparison with non-COPD patients, poorer clinical outcomes as a consequence of their reduced lung function and oxygenation, a higher inflammatory state, a greater extent of arteriosclerosis, and more cardiovascular risk factors. In addition, they have a lower probability of receiving secondary prevention drugs after any MI and life-saving interventions, including PCI at the time of initial presentation or during hospitalization [23,24], as previously described in our study. In this way, in a large population-based study in the United Kingdom (UK), the 6 month mortality rates were significantly higher among COPD patients with MI, both non-STEMI and STEMI, compared to those without COPD [12]. Similar results were found in further studies reporting doubled mortality rates for COPD patients compared to non-COPD patients at follow-up [23,25]. In our study, IHM in STEMI and NSTEMI patients showed significantly higher figures in COPD men than in those without COPD after matching. However, we did not find differences among women, possibly due to the small sample size.

In previous studies, age, female gender, and STEMI were identified as important determinants of survival and mortality in MI patients [26,27]. In our study, IHM in men and women with COPD also increased with age, in addition to other factors such as congestive heart failure, dementia, acute and chronic respiratory failure, and the need for mechanical ventilation during the hospitalization. On the other hand, renal diseases, atrial fibrillation, and pulmonary hypertension were only risk factors for IHM in men suffering COPD. By contrast, undergoing a PCI was associated with a reduction in IHM in all study groups. Invasive management in patients with STEMI and NSTEMI was previously associated with lower mortality, compared with noninvasive management [28,29].

As previously described [30], we found that STEMI was more lethal than NSTEMI. In this way, the probability of dying in COPD men was 2.33 times higher among those with STEMI than among those with NSTEMI. Among women with COPD, this risk was even higher.

Among men and after controlling for study variables, we found that suffering COPD increased IHM after an MI by 14%. Women with COPD had a significantly higher probability of dying in the hospital than COPD men. Sidney et al. [31] also found that female patients had a higher risk of mortality when they studied the relationship between COPD and cardiovascular disease hospitalizations. Sex-related differences in the management and outcomes of acute coronary syndromes were reported in previous studies [32]. Women may have a greater burden of comorbidities and are more likely than men to have atypical symptoms [33]. In addition, there are concerns that women less frequently receive optimal treatment according to the current guidelines [34], including invasive procedures [35]. Furthermore, they also experience more adverse events such as major bleeding and vascular access-related complications [33].

Our study had several limitations related to the nature of this database, including the potential for errors in coding. In addition, it lacked information on some variables of interest, such as pulmonary function test results, medication, or cause of death. Unfortunately, smoking/vaping is a condition that is not collected by the SNHDD, as this database collects only those diagnoses, present at admission or that appear afterward, that are a cause for the patient’s hospitalization or that result in this hospitalization being longer or more complicated than expected. However, the main strength of this investigation is that sample size was large and representative of a national population. Thus, it included all hospital admissions for MI in Spain, providing an unequaled statistical power to examine the relationship between COPD and characteristics and in-hospital outcomes in patients with STEMI and NSTEMI.

## 5. Conclusions

In this large, nationwide, population-based study, we demonstrated that incidence of STEMI and NSTEMI was higher in men and women with COPD than in those without COPD, with the incidence being higher in COPD men than in COPD women. IHM was significantly higher in COPD men than in those without COPD, but no differences were found among women. Suffering COPD increased IHM after an MI by 14% in men. Women with COPD had a significantly higher probability of dying in the hospital than COPD men. Our findings could be taken into account when planning future actions to improve the treatment and care these patients receive.

## Figures and Tables

**Table 1:** Incidence of myocardial infarction (MI) with and without ST elevation, according to the presence of COPD, sex, and age group.

		Men			Women			Both		
		COPD	No COPD		COPD	No COPD		COPD	No COPD	
MI Type	Age Group	*N* (Inc/10^5^)	*N* (Inc/10^5^)	*p*-Value	*N* (Inc/10^5^)	*N* (Inc/10^5^)	*p*-Value	*N* (Inc/10^5^)	*N* (Inc/10^5^)	*p*-Value
STEMI	40–59 years	543 (92.61)	25,483 (121.03)	<0.001	95 (24.39)	4544 (21.35)	0.198	638 (65.38)	30,027 (70.92)	0.042
	60–69 years	950 (193.46)	15,125 (225.17)	<0.001	167 (91.85)	4030 (56.73)	<0.001	1117 (166)	19,155 (138.59)	<0.001
	70–79 years	1191 (198.66)	10,836 (244.41)	<0.001	160 (103.82)	5161 (95.44)	0.294	1351 (179.27)	15,997 (162.56)	0.005
	≥80 years	1235 (248.9)	7804 (319.52)	<0.001	255 (385.94)	8917 (201.7)	<0.001	1490 (265.01)	16,721 (243.63)	0.001
	All age groups	3919 (180.34)	59,248 (171)	0.001	677 (85.53)	22,652 (59.27)	<0.001	4596 (155.03)	81,900 (112.4)	<0.001
NSTEMI	40–59 years	326 (55.6)	12,144 (57.68)	0.513	69 (17.71)	2763 (12.98)	0.010	395 (40.48)	14,907 (35.21)	0.006
	60–69 years	907 (184.7)	10,620 (158.1)	<0.001	154 (84.7)	3238 (45.58)	<0.001	1061 (157.68)	13,858 (100.26)	<0.001
	70–79 years	1528 (254.87)	10,264 (231.51)	<0.001	156 (101.22)	5364 (99.2)	0.803	1684 (223.45)	15,628 (158.81)	<0.001
	≥80 years	1623 (327.1)	9180 (375.86)	<0.001	217 (328.43)	9299 (210.34)	<0.001	1840 (327.26)	18,479 (269.24)	<0.001
	All age groups	4384 (201.74)	42,208 (121.82)	<0.001	596 (75.29)	20,664 (54.07)	<0.001	4980 (167.98)	62,872 (86.28)	<0.001
Total	40–59 years,	869 (148.22)	37,627 (178.71)	<0.001	164 (42.1)	7307 (34.33)	0.009	1033 (105.86)	44,934 (106.12)	0.936
	60–69 years	1857 (378.15)	25,745 (383.27)	0.575	321 (176.54)	7268 (102.3)	<0.001	2178 (323.68)	33,013 (238.85)	<0.001
	70–79 years	2719 (453.54)	21,100 (475.92)	0.017	316 (205.04)	10,525 (194.64)	0.361	3035 (402.72)	31,625 (321.36)	<0.001
	≥80 years	2858 (576)	16,984 (695.38)	<0.001	472 (714.38)	18,216 (412.04)	<0.001	3330 (592.26)	35,200 (512.87)	<0.001
	All age groups	8303 (382.09)	101,456 (292.82)	<0.001	1273 (160.82)	43,316 (113.34)	<0.001	9576 (323.01)	144,772 (198.68)	<0.001

COPD, chronic obstructive pulmonary disease; Inc/10^5^, incidence per 100,000 people with or without COPD; STEMI, ST elevation myocardial infarction; NSTEMI, non-ST elevation myocardial infarction. The *p*-values for comparison of the incidence between patients with and without COPD were adjusted by age and sex using Poisson regression when required.

**Table 2:** Clinical characteristics and hospital outcomes before and after matching by age and myocardial infarction type (International Classification of Disease version 10, ICD-10) for men with and without COPD who suffered an STEMI.

STEMI-Related Variables	Without Matching			After Matching		
	COPD	No COPD	*p*-Value	COPD	No COPD	*p*-Value
Left main CA, *n* (%)	22 (0.56)	343 (0.58)	0.999	22 (0.56)	22 (0.56)	NA
Left anterior descending CA, *n* (%)	407 (10.39)	7915 (13.36)	<0.001	407 (10.39)	407 (10.39)	NA
Other CA of anterior wall, *n* (%)	866 (22.1)	14,006 (23.64)	<0.001	866 (22.1)	866 (22.1)	NA
Right coronary artery, *n* (%)	543 (13.86)	9054 (15.28)	<0.001	542 (13.83)	542 (13.83)	NA
Other CA of inferior wall, *n* (%)	1075 (27.43)	16,626 (28.06)	<0.001	1075 (27.44)	1075 (27.44)	NA
Left circumflex CA, *n* (%)	75 (1.91)	1412 (2.38)	0.101	75 (1.91)	75 (1.91)	NA
Other sites, *n* (%)	232 (5.92)	3324 (5.61)	0.095	232 (5.92)	232 (5.92)	NA
Unspecified site, *n* (%)	699 (17.84)	6568 (11.09)	<0.001	699 (17.84)	699 (17.84)	NA
Age, mean (SD)	72.59 (10.9)	63.4 (12.54)	<0.001	72.59 (10.9)	72.58 (10.9)	NA
CCI, mean (SD)	1.94 (1)	1.57 (0.81)	<0.001	1.94 (1)	1.76 (0.9)	<0.001
Obesity, *n* (%)	499 (12.73)	6836 (11.54)	0.024	499 (12.74)	371 (9.47)	<0.001
Diabetes, *n* (%)	1224 (31.23)	14,519 (24.51)	<0.001	1223 (31.21)	1153 (29.43)	0.085
Hypertension, *n* (%)	1887 (48.15)	25,492 (43.03)	<0.001	1886 (48.14)	1864 (47.58)	0.619
Lipid metabolism disorders, *n* (%)	1783 (45.5)	26,494 (44.72)	0.342	1783 (45.51)	1737 (44.33)	0.296
Renal disease, *n* (%)	565 (14.42)	3666 (6.19)	<0.001	565 (14.42)	394 (10.06)	<0.001
Atrial fibrillation, *n* (%)	716 (18.27)	5676 (9.58)	<0.001	715 (18.25)	621 (15.85)	0.005
Congestive heart failure, *n* (%)	854 (21.79)	7258 (12.25)	<0.001	853 (21.77)	654 (16.69)	<0.001
Peripheral vascular disease, *n* (%)	390 (9.95)	2660 (4.49)	<0.001	390 (9.95)	231 (5.9)	<0.001
Cerebrovascular disease, *n* (%)	139 (3.55)	1441 (2.43)	<0.001	139 (3.55)	141 (3.6)	0.903
Dementia, *n* (%)	81 (2.07)	514 (0.87)	<0.001	81 (2.07)	57 (1.45)	0.039
Dependence on oxygen, *n* (%)	157 (4.01)	146 (0.25)	<0.001	157 (4.01)	6 (0.15)	<0.001
Respiratory failure, *n* (%)	418 (10.67)	1720 (2.9)	<0.001	418 (10.67)	148 (3.78)	<0.001
Sleep apnea, *n* (%)	407 (10.39)	2343 (3.95)	<0.001	407 (10.39)	170 (4.34)	<0.001
Pulmonary hypertension, *n* (%)	100 (2.55)	573 (0.97)	<0.001	100 (2.55)	55 (1.4)	<0.001
Mechanical ventilation, *n* (%)	371 (9.47)	3568 (6.02)	<0.001	371 (9.47)	262 (6.69)	<0.001
CABG, *n* (%)	41 (1.05)	675 (1.14)	0.594	41 (1.05)	55 (1.4)	0.151
PCI, *n* (%)	2131 (54.38)	38,537 (65.04)	<0.001	2131 (54.39)	2265 (57.81)	0.002
LOHS, median (IQR)	5 (6)	5 (4)	<0.001	5 (6)	5 (4)	<0.001
In-hospital mortality, *n* (%)	453 (11.56)	3551 (5.99)	<0.001	453 (11.56)	392 (10.01)	0.026

CA, coronary artery; NA, not applicable; IQR, interquartile range; COPD, chronic obstructive pulmonary disease; STEMI, ST elevation myocardial infarction; CCI, Charlson comorbidity index; CABG, coronary artery bypass graft; PCI, percutaneous coronary intervention; LOHS, length of hospital stay. The *p*-values for the differences between patients with and without COPD were calculated using Student’s *t*-test, Mann–Whitney test, or chi-square test.

**Table 3:** Clinical characteristics and hospital outcomes before and after matching by age and myocardial infarction type (ICD-10) for women with and without COPD who suffered an STEMI.

STEMI-Related Variables	Without Matching			After Matching		
	COPD	No COPD	*p*-Value	COPD	No COPD	*p*-Value
Left main CA, *n* (%)	6 (0.89)	128 (0.57)	0.672	6 (0.89)	6 (0.89)	NA
Left anterior descending CA, *n* (%)	60 (8.86)	2620 (11.57)	0.004	60 (8.86)	60 (8.86)	NA
Other CA of anterior wall, *n* (%)	154 (22.75)	5632 (24.86)	0.042	154 (22.75)	154 (22.75)	NA
Right coronary artery, *n* (%)	106 (15.66)	2832 (12.5)	<0.001	106 (15.66)	106 (15.66)	NA
Other CA of inferior wall, *n* (%)	173 (25.55)	5647 (24.93)	0.173	173 (25.55)	173 (25.55)	NA
Left circumflex CA *n* (%)	10 (1.48)	358 (1.58)	0.999	10 (1.48)	10 (1.48)	NA
Other sites, *n* (%)	43 (6.35)	1426 (6.3)	0.830	43 (6.35)	43 (6.35)	NA
Unspecified site, *n* (%)	125 (18.46)	4009 (17.7)	0.268	125 (18.46)	125 (18.46)	NA
Age, mean (SD)	73.34 (12.1)	72.95 (13.73)	0.464	73.34 (12.1)	73.34 (12.1)	NA
CCI, mean (SD)	1.91 (0.99)	1.79 (0.91)	0.001	1.91 (0.99)	1.71 (0.88)	<0.001
Obesity, *n* (%)	109 (16.1)	2970 (13.11)	0.024	109 (16.1)	77 (11.37)	0.012
Diabetes, *n* (%)	201 (29.69)	6905 (30.48)	0.659	201 (29.69)	194 (28.66)	0.676
Hypertension, *n* (%)	364 (53.77)	11,586 (51.15)	0.179	364 (53.77)	358 (52.88)	0.744
Lipid metabolism disorders, *n* (%)	322 (47.56)	10,230 (45.16)	0.216	322 (47.56)	332 (49.04)	0.587
Renal disease, *n* (%)	81 (11.96)	2356 (10.4)	0.190	81 (11.96)	66 (9.75)	0.190
Atrial fibrillation, *n* (%)	129 (19.05)	3771 (16.65)	0.098	129 (19.05)	88 (13)	0.002
Congestive heart failure, *n* (%)	184 (27.18)	4678 (20.65)	<0.001	184 (27.18)	125 (18.46)	<0.001
Peripheral vascular disease, *n* (%)	43 (6.35)	688 (3.04)	<0.001	43 (6.35)	15 (2.22)	<0.001
Cerebrovascular disease, *n* (%)	20 (2.95)	892 (3.94)	0.193	20 (2.95)	18 (2.66)	0.742
Dementia, *n* (%)	29 (4.28)	817 (3.61)	0.353	29 (4.28)	25 (3.69)	0.579
Dependence on oxygen, *n* (%)	34 (5.02)	135 (0.6)	<0.001	34 (5.02)	3 (0.44)	<0.001
Respiratory failure, *n* (%)	94 (13.88)	1186 (5.24)	<0.001	94 (13.88)	41 (6.06)	<0.001
Sleep apnea, *n* (%)	41 (6.06)	395 (1.74)	<0.001	41 (6.06)	15 (2.22)	<0.001
Pulmonary hypertension, *n* (%)	27 (3.99)	717 (3.17)	0.230	27 (3.99)	14 (2.07)	0.039
Mechanical ventilation, *n* (%)	63 (9.31)	1365 (6.03)	<0.001	63 (9.31)	43 (6.35)	0.043
CABG, *n* (%)	9 (1.33)	135 (0.6)	0.016	9 (1.33)	4 (0.59)	0.163
PCI, *n* (%)	337 (49.78)	11,363 (50.16)	0.844	337 (49.78)	351 (51.85)	0.447
LOHS, median (IQR)	6 (6)	5 (5)	0.001	6 (6)	5 (5)	0.097
In-hospital mortality, *n* (%)	102 (15.07)	2942 (12.99)	0.114	102 (15.07)	89 (13.15)	0.310

CA, coronary artery; NA, not applicable; IQR, interquartile range; COPD, chronic obstructive pulmonary disease; STEMI, ST elevation myocardial infarction; CCI, Charlson comorbidity index; CABG, coronary artery bypass graft; PCI, percutaneous coronary intervention; LOHS, length of hospital stay. The *p*-values for the differences between patients with and without COPD were calculated using Student’s *t*-test, Mann–Whitney test, or chi-square test.

**Table 4:** Clinical characteristics and hospital outcomes before and after matching by age and myocardial infarction type (ICD-10) for men with and without COPD who suffered an NSTEMI.

	Without Matching			After Matching		
Variables	COPD	No COPD	*p*-Value	COPD	No COPD	*p*-Value
NSTEMI, *n* (%)	4384 (100)	42,208 (100)	<0.001	4384 (100)	4384 (100)	NA
Age, mean (SD)	74.81 (9.75)	67.84 (12.63)	<0.001	74.81 (9.75)	74.81 (9.75)	NA
CCI, mean (SD)	2.27 (1.12)	1.84 (0.99)	<0.001	2.27 (1.12)	2.02 (1.06)	<0.001
Obesity, *n* (%)	628 (14.32)	5403 (12.8)	0.004	628 (14.32)	453 (10.33)	<0.001
Diabetes, *n* (%)	1785 (40.72)	14,704 (34.84)	<0.001	1785 (40.72)	1711 (39.03)	0.107
Hypertension, *n* (%)	2102 (47.95)	20,919 (49.56)	0.042	2102 (47.95)	2270 (51.78)	<0.001
Lipid metabolism disorders, *n* (%)	2254 (51.41)	21,914 (51.92)	0.524	2254 (51.41)	2261 (51.57)	0.881
Renal disease, *n* (%)	982 (22.4)	5574 (13.21)	<0.001	982 (22.4)	793 (18.09)	<0.001
Atrial fibrillation, *n* (%)	967 (22.06)	5596 (13.26)	<0.001	967 (22.06)	801 (18.27)	<0.001
Congestive heart failure, *n* (%)	1196 (27.28)	6374 (15.1)	<0.001	1196 (27.28)	845 (19.27)	<0.001
Peripheral vascular disease, *n* (%)	697 (15.9)	3743 (8.87)	<0.001	697 (15.9)	479 (10.93)	<0.001
Cerebrovascular disease, *n* (%)	270 (6.16)	1590 (3.77)	<0.001	270 (6.16)	217 (4.95)	0.013
Dementia, *n* (%)	85 (1.94)	454 (1.08)	<0.001	85 (1.94)	73 (1.67)	0.335
Dependence on oxygen, *n* (%)	251 (5.73)	178 (0.42)	<0.001	251 (5.73)	23 (0.52)	<0.001
Respiratory failure, *n* (%)	535 (12.2)	1399 (3.31)	<0.001	535 (12.2)	213 (4.86)	<0.001
Sleep apnea, *n* (%)	528 (12.04)	2260 (5.35)	<0.001	528 (12.04)	210 (4.79)	<0.001
Pulmonary hypertension, *n* (%)	141 (3.22)	714 (1.69)	<0.001	141 (3.22)	105 (2.4)	0.020
Mechanical ventilation, *n* (%)	279 (6.36)	1491 (3.53)	<0.001	279 (6.36)	187 (4.27)	<0.001
CABG, *n* (%)	120 (2.74)	1679 (3.98)	<0.001	120 (2.74)	162 (3.7)	0.011
PCI, *n* (%)	1636 (37.32)	19,891 (47.13)	<0.001	1636 (37.32)	1897 (43.27)	<0.001
LOHS, median (IQR)	6.5 (7)	5 (5)	<0.001	6.5 (7)	6 (5)	<0.001
In-hospital mortality, *n* (%)	299 (6.82)	1609 (3.81)	<0.001	299 (6.82)	252 (5.75)	0.039

COPD, chronic obstructive pulmonary disease; NA, not applicable; IQR, interquartile range; NSTEMI, non-ST elevation myocardial infarction; CCI, Charlson comorbidity index; CABG, coronary artery bypass graft; PCI, percutaneous coronary intervention; LOHS, length of hospital stay. The *p*-values for the differences between patients with and without COPD were calculated using Student’s *t*-test, Mann–Whitney test, or chi-square test.

**Table 5:** Clinical characteristics and hospital outcomes before and after matching by age and myocardial infarction type (ICD-10) for female patients with NSTEMI.

	Without Matching			After Matching		
Variables	COPD	No COPD	*p*-Value	COPD	No COPD	*p*-Value
NSTEMI, *n* (%)	596 (100)	20,664 (100)	0.008	596 (100)	596 (100)	NA
Age, mean (SD)	73.86 (11.54)	75.31 (12.41)	0.005	73.86 (11.54)	73.86 (11.54)	NA
CCI, mean (SD)	2.05 (0.99)	1.97 (1)	0.054	2.05 (0.99)	1.93 (0.97)	0.036
Obesity, *n* (%)	140 (23.49)	3191 (15.44)	<0.001	140 (23.49)	104 (17.45)	0.01
Diabetes, *n* (%)	219 (36.74)	8084 (39.12)	0.241	219 (36.74)	236 (39.6)	0.311
Hypertension, *n* (%)	321 (53.86)	10,986 (53.16)	0.738	321 (53.86)	331 (55.54)	0.561
Lipid metabolism disorders, *n* (%)	300 (50.34)	10,442 (50.53)	0.925	300 (50.34)	313 (52.52)	0.451
Renal disease, *n* (%)	96 (16.11)	3323 (16.08)	0.986	96 (16.11)	84 (14.09)	0.332
Atrial fibrillation, *n* (%)	144 (24.16)	4083 (19.76)	0.008	144 (24.16)	100 (16.78)	0.002
Congestive heart failure, *n* (%)	180 (30.2)	4705 (22.77)	<0.001	180 (30.2)	122 (20.47)	<0.001
Peripheral vascular disease, *n* (%)	42 (7.05)	960 (4.65)	0.006	42 (7.05)	29 (4.87)	0.112
Cerebrovascular disease, *n* (%)	27 (4.53)	939 (4.54)	0.987	27 (4.53)	28 (4.7)	0.890
Dementia, *n* (%)	20 (3.36)	688 (3.33)	0.972	20 (3.36)	14 (2.35)	0.296
Dependence oxygen, *n* (%)	56 (9.4)	231 (1.12)	<0.001	56 (9.4)	2 (0.34)	<0.001
Respiratory failure, *n* (%)	98 (16.44)	1254 (6.07)	<0.001	98 (16.44)	34 (5.7)	<0.001
Sleep apnea, *n* (%)	46 (7.72)	598 (2.89)	<0.001	46 (7.72)	13 (2.18)	<0.001
Pulmonary hypertension, *n* (%)	30 (5.03)	806 (3.9)	0.161	30 (5.03)	23 (3.86)	0.325
Mechanical ventilation, *n* (%)	51 (8.56)	735 (3.56)	<0.001	51 (8.56)	16 (2.68)	<0.001
CABG, *n* (%)	13 (2.18)	373 (1.81)	0.498	13 (2.18)	12 (2.01)	0.840
PCI, *n* (%)	196 (32.89)	6795 (32.88)	0.999	196 (32.89)	209 (35.07)	0.427
LOHS, median (IQR)	6 (7)	6 (5)	0.003	6 (7)	6 (6)	0.000
In-hospital mortality, *n* (%)	48 (8.05)	1372 (6.64)	0.173	48 (8.05)	34 (5.7)	0.109

COPD, chronic obstructive pulmonary disease; NA, not applicable; IQR, interquartile range; NSTEMI, non-ST elevation myocardial infarction; CCI, Charlson comorbidity index; CABG, coronary artery bypass graft; PCI, percutaneous coronary intervention; LOHS, length of hospital stay. The *p*-values for the differences between patients with and without COPD were calculated using Student’s *t*-test, Mann–Whitney test, or chi-square test.

**Table 6:** Logistic regression analysis to identify variables associated with in-hospital mortality (IHM) among patients with and without COPD, according to sex.

	Men			Women		
	COPD	No COPD	Both	COPD	No COPD	Both
40–59 years	1	1	1	1	1	1
60–69 years	1.47 (0.92–2.35)	2.68 (1.36–5.27)	1.84 (1.26–2.7)	3.17 (1.04–9.72)	1.13 (0.39–3.24)	1.99 (0.94–4.19)
70–79 years	2.38 (1.53–3.7)	4.71 (2.46–8.99)	3.06 (2.13–4.4)	2.86 (0.94–8.7)	1.23 (0.44–3.42)	1.88 (0.9–3.95)
≥80 years	4.12 (2.66–6.38)	10.05 (5.29–19.09)	5.8 (4.05–8.3)	6.25 (2.13–18.33)	3.16 (1.21–8.22)	4.48 (2.22–9.05)
Obesity	NS	NS	0.77 (0.64–0.98)	NS	NS	0.74 (0.61–0.86)
Diabetes	NS	NS	NS	NS	1.36 (1.01–1.69)	1.26 (1.06–1.47)
Renal diseases	1.41 (1.14–1.74)	1.30 (1.07–1.53)	1.34 (1.16–1.58)	NS	NS	1.24 (1.02–1.42)
Atrial fibrillation	1.34 (1.12–1.62)	1.23 (1.01–1.49)	1.29 (1.12–1.49)	NS	1.28 (1.07–2.50)	1.17 (1.06–1.28)
Congestive heart failure	1.32 (1.1–1.58)	1.69 (1.38–2.08)	1.47 (1.29–1.69)	1.55 (1.06–2.28)	1.43 (1.08–1.68)	1.39 (1.09–1.59)
Peripheral vascular disease	NS	1.42 (1.07–1.89)	1.22 (1.02–1.45)	NS	1.64 (1.14–1.94)	1.49 (1.5–1.85)
Cerebrovascular disease	NS	1.59 (1.18–2.49)	1.39 (1.11–1.75)	NS	1.92 (1.35–2.45)	1.72 (1.06–2.30)
Dementia	2.06 (1.38–3.08)	3.01 (1.95–4.65)	2.44 (1.82–3.27)	2.34 (1.19–4.62)	2.18 (1.01–4.7)	2.26 (1.38–3.75)
Acute and chronic respiratory failure	1.92 (1.56–2.36)	2.2 (1.64–2.95)	1.99 (1.68–2.36)	1.64 (1.03–2.63)	2.46 (1.29–4.7)	1.86 (1.28–2.71)
Pulmonary hypertension	1.47 (1–2.16)	NS	1.37 (1.02–1.83)	NS	NS	NS
Mechanical ventilation	7.75 (6.26–9.6)	12.23 (9.44–15.86)	9.32 (7.91–10.99)	4.89 (2.89–8.28)	6.99 (3.57–13.71)	5.88 (3.91–8.86)
CABG	NS	0.63 (0.46–0.97)	0.58 (0.4–0.87)	NS	NS	NS
PCI	0.44 (0.36–0.53)	0.33 (0.27–0.41)	0.39 (0.34–0.44)	0.44 (0.28–0.71)	0.31 (0.18–0.52)	0.38 (0.27–0.53)
STEMI/NSTEMI	2.33 (1.96–2.77)	2.48 (2.05–3)	2.4 (2.12–2.73)	2.63 (1.75–3.95)	3.36 (2.08–5.41)	2.85 (2.1–3.87)
COPD	NA	NA	1.14 (1.03–1.27)	NA	NA	1.06 (0.93–1.38)

COPD, chronic obstructive pulmonary disease; STEMI, ST elevation myocardial infarction; NSTEMI, non-ST elevation myocardial infarction; CABG, coronary artery bypass graft; PCI, percutaneous coronary intervention; NA, not applicable; NS, not significant.

## Data Availability

All investigators can ask the Spanish Ministry of Health for access to the SNHDD, which is provided free of charge, at the following site: https://www.mscbs.gob.es/estadEstudios/estadisticas/estadisticas/estMinisterio/SolicitudCMBDdocs/2018_Formulario_Peticion_Datos_RAE_CMBD.pdf (accessed 12 November 2020).

## Supplementary Materials

The following are available online at https://www.mdpi.com/2077-0383/10/4/652/s1: Table S1. International Classification of Disease version 10 (ICD-10) codes for the clinical diagnosis and procedures used in this investigation; Table S2. Distribution of men and women with and without COPD according to myocardial infarction type and age; Table S3. Logistic regression analysis to identify variables associated with in-hospital mortality (IHM) among patients with COPD.
