# Supplementary material for: Sex Differences in the Effects of COPD on Incidence and Outcomes of Patients Hospitalized with ST and Non-ST Elevation Myocardial Infarction: A Population-Based Matched-Pair Analysis in Spain (2016–2018)
Source: J Clin Med. 2021 Feb 8;10(4):652. doi: 10.3390/jcm10040652 (PMC7914459; doi:10.3390/jcm10040652)
Supplement: Supplementary file 1 [file jcm-10-00652-s001.pdf]

Table S1. International Classification of Disease 10th edition (ICD-10) codes for the clinical diagnosis and procedures used in this investigation.

Clinical diagnosis and procedures	ICD-10 codes
STEMI involving left main coronary artery	I21.01
STEMI involving left anterior descending coronary artery	I21.02
STEMI involving other coronary artery of anterior wall	I21.09
STEMI involving right coronary artery	I21.11
STEMI involving other coronary artery of inferior wall	I21.19
STEMI involving left circumflex coronary artery	I21.21
STEMI involving other sites	I21.29
STEMI of unspecified site	I21.3
NSTEMI	I21.4
Obesity	E66.X
Hypertension	I10, I16.6
Lipid metabolism disorders	E78.0X-E78.5
Atrial fibrillation	I48.0, I48.1, I48.2, I48.91
Dependence on supplemental oxygen	Z99.81
Acute and chronic respiratory failure	J96.XX
Sleep apnea	G47.3X
Pulmonary hypertension	I27.2
Mechanical ventilation	5A1945Z, 5A1955Z, 5A1935Z,5A09357, 5A09457, 5A09557
CABG	02100XX, 02110XX, 02120XX, 02130XX
PCI	02703XX, 02713XX, 02723XX, 02733XX

STEMI: ST-elevation myocardial infarction. NSTEMI: non-ST elevation myocardial infarction CABG: Coronary artery bypass graft. PCI: Percutaneous coronary intervention.

Table S2. Distribution of men and women with and without COPD according to myocardial infarction type and age.

	Men			Women		
	COPD	No COPD	P value	COPD	No COPD	P value
STEMI Age, Mean (SD)	72.59(10.9)	63.4(12.54)	<0.001	73.34(12.1)	72.95(13.73)	0.464
STEMI involving left main coronary artery, n(%)	22(0.26)	343(0.34)	0.999	6(0.47)	128(0.3)	0.672
STEMI involving left anterior descending coronary artery, n(%)	407(4.9)	7915(7.8)	<0.001	60(4.71)	2620(6.05)	0.004
STEMI involving other coronary artery of anterior wall, n(%)	866(10.43)	14006(13.8)	<0.001	154(12.1)	5632(13)	0.042
STEMI involving right coronary artery, n(%)	543(6.54)	9054(8.92)	<0.001	106(8.33)	2832(6.54)	<0.001
STEMI involving other coronary artery of inferior wall, n(%)	1075(12.95)	16626(16.39)	<0.001	173(13.59)	5647(13.04)	0.173
STEMI involving left circumflex coronary artery, n(%)	75(0.9)	1412(1.39)	0.1008	10(0.79)	358(0.83)	0.999
STEMI involving other sites, n(%)	232(2.79)	3324(3.28)	0.095	43(3.38)	1426(3.29)	0.830
STEMI of unspecified site, n(%)	699(8.42)	6568(6.47)	<0.001	125(9.82)	4009(9.26)	0.268
ALL STEMI, n (%)	3.919(47.2)	59248(58.4)	<0.001	677(53.18)	226528(52.29)	
NSTEMI Age, Mean (SD)	74.81(9.75)	67.84(12.63)	<0.001	73.86(11.54)	75.31(12.41)	0.005
NSTEMI n(%)	4384(52.8)	42208(41.6)	<0.001	596(46.82)	20664(47.71)	0.008

Table S3. Logistic regression analysis to identify variables associated with in-hospital mortality (IHM) among patients with COPD.

**Variable**	**COPD**
40-59 years	1
60-69 years	1.66(1.08-2.55)
70-79 years	2.42(1.61-3.64)
≥80 years	4.3(2.88-6.44)
Renal diseases	1.35(1.09-1.65)
Atrial fibrillation	1.27(1.07-1.5)
Congestive heart failure	1.38(1.18-1.61)
Dementia	2.11(1.49-2.98)
Acute and chronic respiratory failure	1.87(1.55-2.26)
Pulmonary hypertension	1.32(1.03-1.64)
Mechanical ventilation	7.13(5.85-8.68)
PCI	0.44(0.38-0.52)
STEMI/NSTEMI	2.37(2.03-2.78)
Female sex	1.19(1.01-1.39)
